# Current and emerging therapies for first line treatment of metastatic clear cell renal cell carcinoma

**DOI:** 10.20517/2394-4722.2021.76

**Published:** 2021-07-12

**Authors:** Michael T. Serzan, Michael B. Atkins

**Affiliations:** Georgetown Lombardi Comprehensive Cancer Center, Washington, DC 20057, USA.

**Keywords:** Renal cell carcinoma, nivolumab, ipilimumab, axitinib, pembrolizumab, avelumab, biomarkers

## Abstract

The therapeutic landscape for advanced clear cell renal cell carcinoma (ccRCC) is rapidly evolving with improved knowledge of the biology of disease leading to the incorporation of a variety of antiangiogenic agents and immunotherapies. In this review, we discuss historical, current, and emerging first line treatment options for patients with advanced ccRCC. These include data with single agent vascular endothelial growth factor receptor tyrosine kinase inhibitors (TKIs): sunitinib, pazopanib and cabozantinib as well as the recently reported results for the combination of lenvatinib and everolimus (mTOR inhibitor). We also discuss results of the nivolumab anti-programmed cell death (PD-1)/ipilimumab (anti-cytotoxic T lymphocyte-associated antigen 4) combination as well as emerging front-line data with nivolumab and pembrolizumab (anti-PD-1) monotherapy. Finally, we review data supporting recent approvals of TKI and anti-PD-1 or anti-PD-Ligand 1 (PD-L1) combinations (e.g., axitinib/pembrolizumab, axitinib/avelumab and cabozantinib/nivolumab) and initial outcomes of lenvatinib (multi-kinase inhibitor) and pembrolizumab. With many individual and combination treatment options and the lack of head-to-head comparisons, treatment selection will depend on the goals of therapy (endpoints) and the identification and validation of clinical and tumor-based predictive biomarkers that are linked to the desired treatment endpoints.

## INTRODUCTION

Kidney cancer is increasing in incidence worldwide with 403,000 new cases and 175,000 deaths annually based on the most recent GLOBOCAN statistics from 2018^[1]^. Renal cell carcinoma (RCC) is the most common form of kidney cancer, and is further classified by histologic subtypes with clear cell (cc) RCC being the most common (75%) followed by papillary (10%) and chromophobe (5%)^[2]^. Localized RCC is typically managed with partial or radical nephrectomy associated with 5-year survival rates ranging from 70% to 90% depending on stage; however, up to 20% of such patients experience metastatic recurrence^[3]^. Approximately 20% of patients are diagnosed with metastatic disease at initial presentation. Metastatic clear cell renal cell carcinoma (ccRCC) historically carried a 5-year survival rate of 13%^[4]^. Advances in understanding the pathophysiology of RCC have elucidated the roles for targeted therapy against vascular endothelial growth factor receptors (VEGFR) with multi-kinase inhibitors, immune checkpoint inhibitors (ICI), and combination anti-VEGF and ICI regimens that have markedly improved outcomes.

ccRCC is near ubiquitously characterized by loss of heterozygosity of the von Hippel Lindau (*VHL*) gene (90%) on chromosome 3p8 due to *VHL* gene mutation (82%) or epigenetic hypermethylation (8%)^[5–7]^. Functional inactivation of the VHL tumor suppressor gene leads to accumulation of the transcription factor Hypoxia Inducible Factor-2α in the absence of hypoxia. This accumulation serves as an oncoprotein driving several downstream pathways including *VEGFA* production leading to highly vascularized tumors^[8,9]^. Anti-angiogenic tyrosine kinase inhibitors (TKI) of the VEGF pathway including sunitinib, pazopanib, and cabozantinib have improved outcomes in randomized clinical trials and are FDA approved therapies in the first line setting for metastatic RCC^[10–13]^. The Cancer Genome Atlas comprehensive genetic analyses have identified a subset of ccRCC patients with alterations in genes including *MTOR* (6%), *PTEN* (4.3%), and *PIK3CA* (2.9%), leading to activation of the mechanistic target of rapamycin (mTOR) pathway^[6]^. Activation of this intracellular pathway leads to increased cell growth and division, thereby presenting biologic rationale for mTOR inhibition with everolimus and temsirolimus^[14]^.

Immunotherapy with checkpoint inhibitors (ICI) to the programmed cell death (PD-1) and cytotoxic T lymphocyte-associated antigen 4 (CTLA-4) pathways have also been investigated in patients with ccRCC. Nivolumab (anti-PD1) was approved in the second line for patients whose disease had progressed on anti-angiogenic therapy based on the phase 3 Checkmate 025 study demonstrating overall survival (OS) and overall response rate (ORR) benefits compared to the mTOR inhibitor, everolimus^[15]^. The Checkmate 214 trial compared combination nivolumab and ipilimumab (anti-CLTA-4) to sunitinib for patients with treatment naïve advanced RCC. This study demonstrated significant improvement in OS and ORR favoring combination ICI therapy in the intermediate/poor risk (see below) populations, leading to FDA approval in April 2018^[16]^.

In this review, we discuss the recent and emerging first-line treatment options in ccRCC, with a focus on axitinib/pembrolizumab, axitinib/avelumab, cabozantinib/nivolumab, and lenvatinib/pembrolizumab and compare their efficacy to nivolumab/ipilimumab as well as VEGFR TKI and CPI monotherapy. We review safety and efficacy data and provide treatment recommendations based on clinical evidence and desired goals of therapy. In addition, we consider treatment sequencing and the need for biomarkers; we look to the future as novel combinations with immunotherapy backbones come to the forefront of the treatment paradigm.

### Clinical prognostic biomarkers

The selection of first line treatment for patients with advanced ccRCC has been guided by risk stratification models developed by the Memorial Sloan Kettering Cancer Center (MSKCC) and the International Metastatic Renal Cell Carcinoma Database Consortium (IMDC)^[17,18]^. The earlier MSKCC model was developed to predict benefit from interferon-α, whereas the IMDC model predicted benefit from VEGFR TKI. Both models include time from diagnosis to treatment, Karnofsky performance status, and hemoglobin and calcium concentrations. Additionally, the MSKCC model incorporates lactate dehydrogenase level, whereas IMDC includes neutrophil and platelet count. In both models, patients with favorable-risk disease have 0 risk factors, those with intermediate-risk disease have 1–2 factors, and those with poor-risk disease have greater than 3 factors. The IMDC model has been utilized as a risk stratification tool for clinical trials of VEGFR TKI and combination regimens; however, its applicability to immunotherapy is likely limited. We will highlight potential future mostly laboratory biomarkers in development.

## FIRST LINE TREATMENT OPTIONS FOR CCRCC

### Targeting angiogenesis and the VEGF pathway

ccRCC is strongly associated with mutations in the *VHL* tumor suppressor gene, which results in functional inactivation of VHL proteins and downstream hypoxia-independent upregulation of pro-angiogenic factors including VEGF. Among all epithelial cancers, ccRCC has the highest expression of *VEGFA*, providing rationale for targeting VEGF and its receptor^[19]^. VEGF receptor blockade with TKIs in RCC has demonstrated several physiologic changes including reduction in blood vessel density, decreased tumor perfusion, and may lead to infarction of the VEGF-dependent tumor microenvironment^[20]^. Resistance to TKI therapy has been demonstrated to occur by angiogenic escape through activation of compensatory vascular signaling pathways including platelet derived growth factor (PDGFR), MET, AXL, and fibroblast growth factor receptor (FGFR)^[21]^. TKIs to multiple tyrosine kinases in addition to the VEGF receptor, including PDGFR (pazopanib, sunitinib, lenvatinib), MET/AXL (cabozantinib), FGFR (lenvatinib) were developed to simultaneously target parallel pathways causing decreased tumor vascularization and growth and delayed angiogenic escape^[22]^.

Sunitinib and pazopanib were the first TKI to improve PFS in the first line setting compared to interferon-alpha (IFNα) and placebo, respectively^[11,12]^. Sunitinib was FDA approved for first line therapy of ccRCC in January 2006 followed by pazopanib in October 2009. These agents were compared in the phase 3 COMPARZ trial with pazopanib demonstrating noninferiority in PFS with similar OS in all IMDC risk groups^[12]^. However, there were considerable differences in OS outcomes for each agent across IMDC groups: favorable risk 42.5 and 43.6 months, intermediate risk 26.9 and 26.1 months, and poor risk 9.9 and 7.7 months, respectively. Patients required frequent dose reductions (44%−51%) and discontinuation (20%−24%) due to adverse effects with similar grade 3–4 hypertension (15%). Differences in safety and tolerability were noted with pazopanib demonstrating higher rates of liver function abnormalities and sunitinib higher rates of fatigue, palmar-plantar dysesthesia, and cytopenias. The phase 3b PISCES sequential cross-over trial demonstrated superior patient and provider preference as well as higher health-related quality of life measures for pazopanib over sunitinib^[23]^. Pazopanib emerged as a preferred front line VEGFR TKI agent based on similar efficacy, less toxicity, and better tolerability.

Although the mTOR inhibitor temsirolimus was approved in the first line setting for patients with intermediate and poor risk RCC based on its superiority to interferon in the Global ARCC trial, the RECORD-3 trial subsequently showed that the oral mTOR inhibitor everolimus was inferior to sunitinib across all IMDC risk groups^[24,25]^. As a consequence, mTOR inhibitor use has been relegated to second or later lines of therapy, particularly in patients with tumors showing mutations in the PI3K, MTOR, TSC pathway or in combination with VEGFR TKI such as lenvatinib^[26]^.

*MET* and *AXL* expression has been associated with aggressive disease and may mediate resistance to VEGFR TKI therapy^[27]^. The randomized phase 2 CABOSUN trial compared cabozantinib, an oral multi kinase inhibitor to VEFGR, MET, and AXL to sunitinib for treatment-naïve patients with intermediate/poor risk disease^[13]^. The trial met its primary endpoint of investigator assessed PFS (HR = 0.66; 95%CI: 0.46–0.95; *P* = 0.012) which was confirmed by independent radiology committee (IRC) with extended follow up (HR = 0.48; 95%CI: 0.31–0.74, *P* = 0.0008)^[28]^. Further analysis demonstrated PFS benefit of cabozantinib over sunitinib across both IMDC risk groups, and regardless of tumor burden, metastatic site and *MET* expression status. Although there was a trend towards longer overall survival with cabozantinib 26.6 months *vs*. sunitinib 21.2 months (HR = 0.80; 95%CI: 0.53–1.21), this study was underpowered to assess OS differences. Cabozantinib tolerance was similar to sunitinib with comparable rates of dose reduction (46% *vs*. 35%) and discontinuation (21% *vs*. 22%). Also, common grade 3–4 adverse events were similar between cabozantinib and sunitinib with hypertension (28% *vs*. 21%), fatigue (6% *vs*. 17%), diarrhea (10% *vs*. 11%), and thrombocytopenia (1% *vs*. 11%). Based on the PFS benefit, cabozantinib was approved by the FDA in December 2017 for patients with intermediate/poor risk treatment naïve ccRCC.

Most recently, the phase 3 CLEAR study for patients with all-risk ccRCC compared first line sunitinib to lenvatinib/everolimus or lenvatinib/pembrolizumab^[29]^. The study met its primary endpoint of PFS by IRC for lenvatinib/everolimus compared to sunitinib (HR = 0.65; 95%CI: 0.53–0.80). Despite this PFS benefit with higher ORR (54% *vs* 36%) and CR rates (10% *vs* 4%), there was no difference in overall survival (HR = 1.15; 95%CI: 0.88–1.50). These results suggest that either the FGFR inhibition from lenvatinib or the addition of mTOR inhibition with everolimus may lead to enhanced initial antitumor response; however, this benefit may compromise the efficacy of subsequent therapy, thereby limiting the impact of this regimen on OS.

Despite the PFS benefits of sunitinib, pazopanib, cabozantinib, and lenvatinib/everolimus in the first line, these therapies were associated with frequent dose reductions (35%−51%) and rates of discontinuation due to adverse effects (20%−24%), many of which were postulated to be mediated by off-target inhibition of the PDGFR, KIT, and FLT-3 pathways. First line trials utilizing the more potent and selective second generation VEGFR TKIs axitinib and tivozanib compared to sorafenib were hypothesized to improve efficacy and reduce adverse effects. A phase 3 trial of first line axitinib compared to sorafenib showed numerical differences in PFS; however, it did not establish a statistically significant difference between the two treatments 10.1 months *vs*. 6.5 months (HR = 0.77; 0.56–1.05)^[30]^. In addition, while axitinib showed significantly higher ORR 32% *vs*. 15% (1 sided *P* = 0.0006), no difference in median OS 21.7 months *vs*. 23.3 months (HR = 0.95; 0.73–1.36) compared to sorafenib was observed. One key limitation of this study was relatively small sample size (*N* = 288) to detect anything but a large magnitude of difference between therapies. The TIVO-1 study of first line tivozanib, a potent and selective TKI to VEGFR, c-Kit, and PDGFR compared to sorafenib met its primary endpoint of improved median PFS 11.9 months *vs*. 9.1 months (HR = 0.79; 0.63–0.99; *P* = 0.042)^[31]^. However, OS analysis showed a trend toward longer survival on the sorafenib arm than on the tivozanib arm - median 29.3 months *vs*. 28.8 months (HR = 1.245; 0.95–1.62; *P* = 0.105). These discordant PFS and OS results were hypothesized to be related to a greater proportion of patients in the sorafenib arm receiving next-line VEGFR TKI treatment (63% *vs*. 13% in the tivozanib arm) particularly with tivozanib (as part of the study). Although neither axitinib nor tivozanib were approved by the FDA for the first line setting, both of these agents have improved PFS compared to sorafenib in the second or later lines of therapy (leading to their FDA approval) and because of their improved therapeutic index related to their more selective targeting of the VEGF axis, they might offer advantages as backbones for combination regimens^[32,33]^.

Antiangiogenic therapy with TKI greatly improved outcomes for patients with metastatic ccRCC relative to cytokine-based therapies. Despite consistent improvements in ORR and PFS, these regimens were not curative. Patients inevitably experienced progression of disease necessitating sequential switching to a different therapy, often another VEGFR TKI or an mTOR inhibitor. These successive agents increased the cumulative incidence of off-target adverse effects, which impacted quality of life and contributed substantial financial toxicity over time. As such, the current role of antiangiogenic TKI monotherapy is limited to patients who cannot receive ICI therapy due to active autoimmune disease or high dose steroids for central nervous system metastases.

### Immune checkpoint inhibitor therapies

Initial evidence of immunogenicity in RCC was demonstrated in cytokine-based therapies with high dose interleukin 2 and IFNα, which showed durable, complete responses in small subsets of patients^[34,35]^. The ability to induce an adaptive immune response relies on several aspects including tumor antigenicity, extent of immune cell infiltrate and immunomodulatory aspects within the tumor microenvironment^[36]^. ccRCC tumors are characterized by rich leukocyte infiltrates of CD8+ and CD4+ T cells as well as myeloid derived macrophages and neutrophils^[37]^. Tumors with an abundance of myeloid derived suppressor cells (MDSC) and polymorphonuclear leukocytes have been associated with higher tumor grade and shorter overall survival^[38]^.

Checkpoint inhibitors are monoclonal antibodies that block physiologic or tumor cell mediated modulation of cellular immunity thereby restoring antigen specific cytotoxic T cell-mediated immune response^[39]^. Two critical checkpoints include interactions between CTLA receptor and its ligands CD80/86 on antigen presenting cells typically in peripheral immune organs and the PD-1 receptor and its ligands PD-L1/L2 in the tumor microenvironment. CTLA-4 binding to its ligand CD80/86 inhibits T cell activation. Therapeutic inhibition of this interaction with the CTLA-4 inhibitor ipilimumab leads to augmentation of T cell activation and proliferation of T cell subsets^[40]^. In the tumor microenvironment, tumor and immune cells can upregulate PD-L1/L2 ligands that bind the PD-1 receptor on tumor reactive T cells leading to suppression of T cell activity^[41]^. Inhibition of this interaction with PD-1 antibodies (nivolumab, pembrolizumab) or PD-L1 antibodies (avelumab, atezolizumab) restores cytotoxic T cell activity and helper T cell cytokine production.

Nivolumab was the first ICI to show benefit in patients with advanced RCC. Nivolumab was compared to everolimus in patients who had exhibited disease progression on antiangiogenic therapy and showed improved ORR 25% *vs*. 5% and OS 25.0 months *vs*. 19.6 months (HR = 0.73; 0.57–0.93; *P* = 0.002)^[15]^. Activity relative to everolimus was particularly apparent in the MSKCC poor risk population (HR death = 0.47; 0.30–0.73). Although these results were sufficient to confer FDA approval for nivolumab monotherapy, the efficacy was felt to be insufficient to be superior to VEGFR TKIs in treatment naïve patients. However, the Checkmate 016 trial explored the combination of nivolumab and ipilimumab in patients with either treatment naïve or VEGFR TKI resistant ccRCC and showed higher ORR (40.4%) and median PFS (7.7 months) than had been observed with nivolumab monotherapy in the CM 025 trial suggesting it was more efficacious^[42]^.

As a consequence, combination nivolumab and ipilimumab was compared to sunitinib in treatment naïve patients in the Checkmate 214 phase 3 trial with co-primary endpoints OS, PFS, and ORR in the intermediate/poor risk disease groups^[17]^. The study initially met two of three primary endpoints in its target population at median follow up 25.2 months. The combination demonstrated improved OS (HR death = 0.63; *P* < 0.001), ORR 42% *vs*. 27% (*P* < 0.001), and complete response rate 9% *vs*. 1% relative to sunitinib [Table 1]. PFS was improved; however, it did not meet pre-specified level of significance (HR = 0.82; *P* = 0.03). Subgroup analysis confirmed OS and ORR benefit regardless of PD-L1 tumor expression in intermediate/poor risk patients. However, patients with tumor PD-L1 > 1% demonstrated enhanced OS benefit from the combination immunotherapy (HR OS = 0.45; 0.29–0.71) relative to those with tumor PD-L1 < 1% (HR OS = 0.73; 0.56–0.96). Patients with tumor PD-L1 > 1% demonstrated longer PFS with the combination relative to sunitinib (HR PFS = 0.46; 0.31–0.67), whereas patients with tumor PD-L1 < 1% did not (HR PFS = 1.00; 0.80–1.26).

In patients with favorable risk disease, sunitinib demonstrated significantly improved early outcomes relative the nivolumab/ipilimumab with ORR 52% *vs*. 29% (*P* < 0.001) and PFS 25.1 months *vs*. 15.3 months (HR = 2.18; 1.29–3.68; *P* < 0.001) that did not extend to OS (HR = 1.19; *P* = 0.44). Interestingly, the efficacy of nivolumab and ipilimumab was similar in favorable risk patients compared with intermediate/poor risk patients with ORR 39% and 42%, CR 8% and 11.3%, and landmark 42 months PFS 28% and 35%^[43]^. Furthermore, at 48 months there was a crossing of the Kaplan Meier OS curves between sunitinib and nivo/ipi indicating the potential for a OS benefit to eventually emerge favoring the combination^[44]^. Post hoc analysis of patients with aggressive sarcomatoid features, the vast majority of whom had intermediate or poor risk disease, showed remarkable benefits favoring nivolumab/ipilimumab to sunitinib in ORR 60.8% *vs*. 23.1%, CR rate 18.9% *vs*. 3.1%, median PFS 26.5 months *vs*. 5.1 months (HR = 0.54; 0.33–0.86; *P* = 0.0093), and median OS not reached *vs*. 14.2 months (HR = 0.45; 0.3–0.7; *P* = 0.0004)^[45]^. Taken together, nivolumab/ipilimumab has emerged as the standard regimen for patients with intermediate/poor risk disease and those with sarcomatoid features. For patients with favorable risk disease, the early ORR and PFS benefits observed on sunitinib may be attributable to the relative efficacy of anti-angiogenic therapy in patients with less aggressive disease; however, maturing long term data of similar overall survival suggests a potential role for nivolumab/ipilimumab in this population.

Treatment related grade 3–4 adverse events were lower on nivolumab/ipilimumab (46%) compared to sunitinib (63%) [Table 2]. Nivolumab/ipilimumab was associated with high rate of immune related adverse events at 80% with 29% requiring high dose steroids for treatment. The discontinuation rate from all-cause adverse events was 22% for the combination and 13% for sunitinib. Interestingly, patients who discontinued nivolumab/ipilimumab treatment due to toxicity, exhibited a better OS than patients who did not experience treatment limiting toxicity. Patient reported outcomes of quality of life showed consistent mean change from baseline favoring combination therapy relative to sunitinib (*P* < 0.001) which was evident despite discontinuing QOL measurements at the time of treatment discontinuation even in patients continuing to exhibit long-term disease control.

The unprecedented improvements in overall survival with nivolumab/ipilimumab have led to efforts to investigate if subsets of patients could derive similar long-term benefits from single agent anti-PD-1 agents while avoiding toxicity of combination with ipilimumab. The Keynote 427 single-arm phase 2 study examined first line pembrolizumab monotherapy for patients with advanced RCC with the primary endpoint of ORR by blinded independent central review. Patients with ccRCC (Cohort A) demonstrated ORR 36.4% with CR 2.7%, median PFS 7.1 months, and median OS not reached at median follow up 18 months^[46]^. ORR was numerically higher for patients with intermediate/poor risk disease 39.7% compared to favorable risk 31.0% as well as patients with PD-L1 positive tumors 44.2% compared to 29.3% for those with PD-L1 negative tumors. Patients with sarcomatoid differentiation had an ORR of 63.6%. Treatment related adverse effects occurred in 73.6% of patients with grade 3–5 occurring in 18.2%. These results show clinical activity of pembrolizumab monotherapy in the first line setting with perhaps increased benefit in intermediate/poor risk disease and sarcomatoid differentiation groups and lower rates of severe grade 3–5 toxicity relative to nivolumab/ipilimumab.

Similar efforts have been made to investigate both anti-PD-L1 monotherapy in the first line setting and the feasibility of salvage nivolumab/ipilimumab. The HCRN GU 16–260 phase II trial treated patients with advanced ccRCC with first line nivolumab (Part A) with the primary endpoint of ORR^[47]^. Patients who experienced either progression of disease (PD) or stable disease (SD) at 48 weeks were eligible to receive salvage nivolumab/ipilimumab (Part B). In the total population, ORR was 31.7% with CR 5.7% with subgroup analysis showing patients with favorable risk disease had an ORR of 50%, intermediate/poor risk disease had ORR of 25% and those with sarcomatoid tumors had an ORR of 31.8%. The median duration of response was 19.3 months and median PFS was 7.4 months. Sixty patients were potentially eligible for salvage nivolumab/ipilimumab (Part B); however, 28 did not enroll due to symptomatic progression of disease (17), grade 3–4 toxicity on nivolumab (8), or other (3). Of the patients who received salvage therapy, best response was PR (13%), SD (30%), and PD (59%). Grade 3–5 treatment-related adverse effects were seen in 28% on nivolumab monotherapy and 33% on nivolumab/ipilimumab. These results suggest a potential role for anti-PD-1 monotherapy in patients who have contraindications or an aversion to either an ipilimumab or VEGFR TKI containing combination regimen, particularly those with favorable risk disease. However, anti-PD-1 monotherapy is likely inferior to nivolumab/ipilimumab in patients with intermediate/poor risk disease - a question that is being formally addressed in the Checkmate 8Y8 (NCT03873402) protocol which is currently ongoing, or those whose tumors express sarcomatoid features^[48]^.

### Combination VEGF TKI and PD-1 therapy

Angiogenic agents targeting VEGF and ICI therapies have improved survival for patients with advanced ccRCC and are standard therapies in the management of this disease. Combinations of antiangiogenic and ICI therapies have the potential to target distinct and complementary pathways, providing synergistic benefit with concurrent therapy compared with additive effects of sequential therapy. Recent preclinical studies have demonstrated that VEGFR TKI therapy alleviates immunosuppression in the tumor microenvironment through targeting regulatory T-cells and MDSC, promoting T-cell infiltration, and enhancing T-cell mediated cytotoxicity^[49–52]^. *In vivo* evidence from animal models has further shown that combination of sunitinib or cabozantinib with chimeric antigen receptor-modified T cells can increase anti-tumor efficacy and prolong survival compared to immunotherapy alone^[53]^. However, there is also preclinical evidence suggesting that anti-angiogenic therapy may have an antagonistic effect on the immune response, particularly in ccRCC, by increasing hypoxia in the TME thereby diminishing anti-tumor immunity and by upregulating *CXCR4* expression leading to the influx of tumor-infiltrating regulatory T cells and MDSC^[54–56]^.

Early phase I evaluation of combination sunitinib or pazopanib with nivolumab or pembrolizumab for advanced RCC showed high rates of response; however, high-grade toxicities limited further investigation^[57]^. More recently, several trials have investigated various antiangiogenic agents combined with anti-PD1/PD-L1 therapies compared to sunitinib alone. The IMmotion 151 phase 3 trial of first line combination atezolizumab (anti-PD-L1) and bevacizumab (monoclonal antibody against VEGF) was compared to sunitinib with co-primary endpoints PFS in PD-L1+ tumors and OS in overall population^[58]^. In patients with PD-L1+ tumors, the combination demonstrated significantly improved PFS (investigator assessed) compared to sunitinib alone (HR PFS = 0.74; 0.57–0.96; *P* = 0.0217) [Table 1]. In the overall population, there was no significant difference in PFS or OS between atezolizumab plus bevacizumab and sunitinib. On further examination by an IRC, patients with PD-L1+ tumors demonstrated similar PFS between the combination and sunitinib (HS PFS = 0.93; 0.72–1.21). Interestingly, the IRC analysis of patients with PD-L1 negative tumors demonstrated a trend towards longer PFS in the atezolizumab plus bevacizumab groups compared to sunitinib; suggesting that either PD-L1 status is a poor predictive biomarker for response to the combination or that the Ventana SP142 assay scoring immune cell PD-L1 positivity may be a suboptimal assay. Atezolizumab and bevacizumab was well tolerated compared to sunitinib with lower rates of grade 3–4 treatment-emergent adverse events (40% *vs*. 54%) with 16% patients on atezolizumab and bevacizumab requiring corticosteroids for IRAE [Table 2]. Due to discordant PFS between investigator and IRC assessments and the absence of OS benefit, the combination of atezolizumab and bevacizumab was not approved for first line use.

In contrast, two unique combinations of axitinib with either pembrolizumab or avelumab (anti-PD-L1) have demonstrated PFS benefits relative to sunitinib. The Keynote 426 phase 3 trial for first line ccRCC randomized patients to axitinib/pembrolizumab or sunitinib with co-primary endpoints of OS and PFS in the intention-to-treat population^[59]^. At first interim analysis at a median follow up 12.8 months, the combination demonstrated improvements in risk of death (HR OS = 0.53; 0.38–0.74; *P* < 0.0001), risk of progression (HR PFS = 0.69; 0.57–0.84; *P* < 0.001), and ORR (59.3% *vs*. 35.7%; *P* < 0.001) relative to sunitinib [Table 1]. The PFS and OS benefits were observed across all IMDC risk groups and PD-L1 expression categories. In a subsequent analysis at median follow up 27 months, the PFS benefit was maintained (HR = 0.71), but the OS benefit was reduced (HR OS = 0.68; 055–0.85; *P* < 0.001) and was no longer apparent for the favorable risk population (HR OS = 1.06; 0.60–1.86)^[60]^. Axitinib/pembrolizumab had similar rates of grade 3–4 treatment-related adverse events compared to sunitinib at 67% and 62%; however, the combination had higher rates of grade 3–4 liver enzyme elevation 7%−12% *vs*. 2%−3%, respectively [Table 2]. These results led to FDA approval of axitinib plus pembrolizumab for all-risk patients with advanced RCC in April 2019.

Axitinib combined with avelumab was compared to sunitinib in the Javelin Renal 101 phase 3 trial for first line ccRCC with independent primary endpoints of OS and PFS in patients with PD-L1 positive tumors^[61]^. In the PD-L1 positive group, axitinib and avelumab demonstrated improvements in PFS (HR = 0.62; 0.49–0.78; *P* < 0.001) and ORR 55.9% *vs*. 27.2% relative to sunitinib [Table 1]. These benefits of the combination were also demonstrated in the overall study population PFS (HR = 0.69; 0.56–0.84; *P* < 0.001) and ORR 52.5% *vs*. 27.3%^[62]^. Axitinib and avelumab was well tolerated compared to sunitinib with similar rates of grade 3–4 treatment-emergent adverse events (71.2% *vs*. 71.5%) with 11% of combination therapy patients requiring corticosteroids for IRAE [Table 2]. These results led to FDA approval of axitinib plus avelumab for all-risk patients with advanced RCC in May 2019.

Two additional trials utilizing combinations of antiangiogenics with anti-PD-1 agents have resulted within the past year. In the Checkmate9ER phase 3 trial patients with treatment naïve ccRCC were randomized to cabozantinib/nivolumab or sunitinib with the primary endpoint of PFS by blinded independent central review^[63]^. At first interim analysis of median follow up 18.1 months, the combination demonstrated improvements in PFS (HR = 0.51; 0.40–0.64; *P* < 0.0001) and OS (HR = 0.60; 0.40–0.89; *P* < 0.001) relative to sunitinib [Table 1]. The PFS and OS benefits were observed across all IMDC risk groups and *PD-L1* expression categories leading to FDA approval in January 2021. Cabozantinib/nivolumab had similar rates of grade 3–4 treatment-related adverse effects compared to sunitinib at 61% and 51% with 16% of patients on the combination requiring corticosteroids for IRAE [Table 2]. The CLEAR phase 3 trial for first line ccRCC randomized patients to sunitinib, lenvatinib/everolimus, or lenvatinib/pembrolizumab with primary endpoint PFS by IRC per RECIST1.1^[29]^. At first interim analysis at a median follow up of 27 months, lenvatinib/pembrolizumab was superior to sunitinib in PFS (HR = 0.39; 0.32–0.49; *P* < 0.001) and OS (HR = 0.60; 0.40–0.89; *P* = 0.001). Lenvatinib/pembrolizumab also had high rates of grade 3–4 treatment-related adverse events (82%) necessitating dose reduction of lenvatinib in 68% and discontinuation of the combination in 13% of patients. Both cabozantinib/nivolumab and lenvatinib/pembrolizumab achieved high response rates as well as significant PFS and overall survival benefits for the intent to treat population. However, the contribution of cabozantinib and lenvatinib as more efficacious TKIs relative to sunitinib may confound these early results. Furthermore, the ability of these regimens to produce long-term plateauing of the PFS curve as well as their impact on OS over time will be of particular interest given the more limited effective treatment options following disease progression on regimens involving the use of more potent TKIs in the front-line setting.

Combinations of anti-angiogenics with anti-PD-1/PD-L1 have been tolerable with common adverse effects being fatigue, hypertension, and diarrhea similar to adverse effects observed with sunitinib alone. However, higher rates of hypothyroidism (22%−35%) and grade 3–4 liver enzyme elevations (6%−13%) were observed in axitinib/pembrolizumab and axitinib/avelumab than with sunitinib. These warrant close monitoring as antiangiogenic and ICI therapies may potentiate synergistic or additive adverse effects that accumulate over time. Current trials of combination angiogenic and ICI therapies are evaluating patient-reported outcomes to determine in depth health-related quality of life over time while on therapy.

### Novel endpoints

Over the past three years, pivotal trials of anti-PD-1 therapy combined with either anti-CTLA or various VEGFR TKIs have led to unprecedented improvements in outcomes for patients with advanced RCC. However, in the absence of head-to-head comparison, the optimal choice for first line therapy remains a debated issue as these regimens have yielded important differences in some novel endpoints. Anti-PD-1/VEGFR TKI combinations have demonstrated improvements in “early” endpoints including ORR, PFS, and < 2-year OS rates, perhaps favoring use in patients with symptomatic disease with the goal of more immediate disease control. Whereas, anti-PD-1/anti-CTLA therapy has led to more durable “late” endpoints including landmark PFS/OS, > 2-year OS rates, treatment free survival (TFS), and patient-reported quality of life, suggesting greater benefit in sustained disease control.

As data from the Checkmate-214 study continue to mature with a minimum of 4-year follow up, the durable and late benefits have become more pronounced with PFS and OS plateaus greater than 30% and 50%, respectively. The overall survival benefits for the ITT population on nivolumab/ipilimumab relative to sunitinib in the Checkmate 214 study have remained stable over time with initial median follow-up of 25 months (HR OS = 0.68; *P* < 0.001) and interval follow-up at median 43 months (HR OS = 0.72; 0.61–0.86; *P* = 0.0002). Furthermore, Regan *et al*.^[64,65]^ analyzed 42-month TFS, defined as time from protocol therapy cessation to time of subsequent systemic therapy or death. In the nivolumab/ipilimumab arm, 56% of patients were alive, 13% were on nivolumab maintenance, and 31% were surviving free of subsequent therapy. In the sunitinib arm, 47% of patients were alive, 7% remained on sunitinib therapy, and 12% were surviving free of subsequent treatment. The overall 42-month restricted mean TFS was 7.8 months for nivolumab/ipilimumab and 3.3 months for sunitinib. Mean TFS for nivolumab/ipilimumab compared to sunitinib was three times as long in favorable risk patients (11.0 months *vs*. 3.7 months) and over twice as long in intermediate/poor risk patients (6.9 months *vs*. 3.1 months). These results highlight that patients across all IMDC risk groups experienced benefit from ICI therapy with greater survival time treatment-free without toxicity relative to sunitinib. Although nivolumab/ipilimumab carries a high risk of early immune related adverse events, many of these are reversible with short-term immunosuppression without evidence of detriment to anti-tumor immune effect. Based on its ability to produce robust and sustained anti-tumor responses with prolonged treatment free intervals, nivolumab/ipilimumab represents an excellent treatment choice for many patients with advanced ccRCC.

Several anti-PD-1/VEGFR TKI combinations have demonstrated substantial benefit in early endpoints of ORR 52%−71% and median PFS 13–24 months in the ITT populations. However, the ability of these regimens to extend early response benefits into durable long-term outcomes with PFS and OS plateaus remains to be established. Axitinib/pembrolizumab showed remarkable early overall survival benefit in ITT population at a median 12-month follow up (OS HR = 0.53; 0.38–0.74; *P* < 0.001); however, this OS benefit appears to diminish at the median follow-up of 30 months (OS HR = 0.68; 0.55–0.85; *P* = 0.0003) [Table 3]. Similarly, axitinib/avelumab showed early overall survival benefit in ITT at the median follow-up of 12 months (OS HR = 0.69; 0.56–0.84; *P* < 0.001); however, this OS benefit appears to diminish at the median follow-up of 19 months (OS HR = 0.80; 0.61–1.02; *P* = 0.03) [Table 3]. The decreasing OS benefits in these two studies may be due to less robust antitumor activity of the anti-PD-1 backbone compared to nivolumab/ipilimumab and the increasing ability for patients with less aggressive tumors who progress on sunitinib to receive salvage anti-PD1 based immunotherapy. In contrast to the TFS period observed with nivolumab/ipilimumab, there is currently no evidence that anti-PD-1/VEGFR TKI combination regimens can produce continued response after cessation of the VEGFR TKI component. On the other hand, despite the fact that anti-PD-1/VEGFR TKI regimens have high rates of grade 3–4 adverse effects and frequent dose reductions, the Checkmate 9ER trial demonstrated significant improvement in health-related quality of life and burden of symptoms compared with sunitinib^[66]^. As data from several pivotal trials of anti-PD-1/VEGFR TKI therapy continue to mature, their ability to produce improvements in late endpoints such as landmark PFS/OS and treatment free survival relative to nivolumab/ipilimumab will be essential to understanding their role as a front line therapy for patients with advanced ccRCC.

### Predictive biomarkers

Although the MSKCC and IMDC models reliably predicted overall survival in patients receiving TKI therapy, there are currently no validated biomarkers of disease prognosis or prediction of response to ICI therapy or combination TKI/ICI therapy. Tumor PD-L1 expression was initially thought to be a promising biomarker given the observations that it is commonly overexpressed in 23%−56% of ccRCC tumors (depending on the assay) and is associated with poor outcomes^[67,68]^. However, the ability for *PD-L1* expression to reliably predict response to ICI has been inconsistent across studies. In Checkmate 214, patients with PD-L1 positive tumors treated with nivolumab/ipilimumab had improved PFS and OS compared to those with PD-L1 negative tumors. In the Keynote 426 study, patients responded to axitinib/pembrolizumab regardless of tumor PD-L1 status. Lastly, in the IMmotion 151 study patients with PD-L1 negative disease appeared to have the most benefit from atezolizumab/bevacizumab relative to sunitinib. Taken together, PD-L1 positivity appears to predict better ORR on ICI therapy in patients, especially those aggressive disease; however, it remains limited in its ability to predict long-term PFS or OS benefits. *PD-L1* expression appears to also predict lack of benefit from VEGFR TKI therapy alone, confounding its use as a biomarker in anti-PD1/VEGFR TKI combinations.

Acknowledging that most patients with ccRCC have PD-L1 negative tumors, it is important to recognize that the majority of responses to ICI will occur in the PD-L1 negative population. Although its role as a prognostic and predictive biomarker remains unclear, *PD-L1* expression remains an important risk stratification tool for clinical trials. Clearly additional biomarkers predictive of benefit for a particular regimen are needed to help with treatment choices in the current crowded first line therapeutic space.

Perhaps the most promising biomarkers have emerged from extensive genetic and gene expression profiling on tumors from the IMmotion 151 trial. Motzer *et al*.^[69]^ conducted integrated multi-omics evaluation of 823 tumors from patients with ccRCC and identified 7 distinct tumor molecular subsets (1) angiogenic/stromal; (2) angiogenic; (3) complement/oxidation; (4) T-effector/proliferative; (5) proliferative; (6) stromal/proliferative; and (7) SnoRNA^[69]^. The angiogenic groups 1 and 2 appeared to show benefit to both sunitinib and atezolizumab/bevacizumab. The proliferative groups 4–5 as well as group 7 appeared to show greater benefit to atezolizumab/bevacizumab compared to sunitinib alone. Groups 3 and 6 did not appear to benefit from either treatment approach. Lastly, somatic mutations in *PBRM1* and *KDM5C* associate with high angiogenesis and AMPK fatty acid oxidation suggesting benefits from angiogenesis blockade^[70]^. Whereas *CDKN2A*, *BAP1*, and *TP53* appear to associate with increase cell cycle and anabolic metabolism suggesting benefit from ICI therapies^[71]^. Taken together these discoveries, if validated using contemporary FDA approved regimens, could be used to categorize patients based on genetic and gene expression profiles and thus determine the optimal treatment approach (e.g., combination ICI, combination VEGFR TKI/ICI, or novel agents) while also mitigating drug and financial toxicities^[72]^. Importantly, such validation studies for these and other predictive biomarkers should be linked to goals of care including novel endpoints mentioned above such as landmark OS and PFS, TFS and QOL throughout to the TFS period which better reflect the impact of the immunotherapy component.

## FUTURE DIRECTIONS

The role of cytoreductive nephrectomy, which previously provided both symptomatic benefit and improved overall survival in combination with cytokine therapies, is yet to be defined with current ICI and ICI/VEGFR TKI combinations^[73–76]^. The NORDIC-SUN (NCT03977571) and CYTOSHRINK (NCT04090710) trials are evaluating induction nivolumab/ipilimumab followed by delayed cytoreductive nephrectomy or interim stereotactic body radiation, respectively followed by nivolumab maintenance^[77,78]^ [Table 4]. The Phase 2 Cyto-KIK trial (NCT04322955) is evaluating induction nivolumab/cabozantinib with delayed cytoreductive nephrectomy followed by resumption of systemic therapy^[79]^. Lastly, the phase 3 PROBE trial (NCT04510597) includes checkpoint inhibitor-based induction with nivolumab/ipilimumab or axitinib/pembrolizumab followed by randomization to nephrectomy or continuation of systemic therapy for patients with PR/SD and discontinuation of study for patients with CR/PD^[80]^. Each of these studies will collect vital clinicopathologic data that have the potential to our understanding of the biologic processes underlying responses to therapy and guide initial and subsequent treatment choices.

There are several ongoing studies of doublet and triplet regimens for advanced treatment naïve ccRCC. For patients with intermediate/poor risk disease, the phase 3 trials Checkmate 209–8Y8 (NCT03873402) and COSMIC 313 (NCT03937219) are investigating nivolumab/ipilimumab compared to nivolumab alone and nivolumab/ipilimumab/cabozantinib, respectively with primary endpoints of PFS by central review^[54,81]^ [Table 4]. The PD1GREE (NCT03793166) phase 3 adaptive trial for patients with intermediate/poor risk treatment naïve ccRCC compares induction nivolumab/ipilimumab followed by either nivolumab/cabozantinib or nivolumab alone for patients with PR or SD at 12 weeks^[82]^. Following induction nivolumab/ipilimumab, patients with CR continue on nivolumab maintenance, whereas patients with PD change to cabozantinib monotherapy. However, there remains an unmet need for a clinical trial comparing first line nivolumab/ipilimumab to anti-PD1/VEGF therapy for all risk disease patients with endpoints of landmark PFS and OS, complete response rates, treatment free survival, and quality of life. Optimally such a trial could also validate some of the intriguing biomarker data emerging from some of the recently published trials. With a widening landscape of treatment combinations, clinical trials investigating sequences and combinations of therapies must be designed to demonstrate impact on long-term outcomes such as complete responses, landmark PFS/OS, treatment free survival, and quality of life.

## CONCLUSION

**Figure 1.**
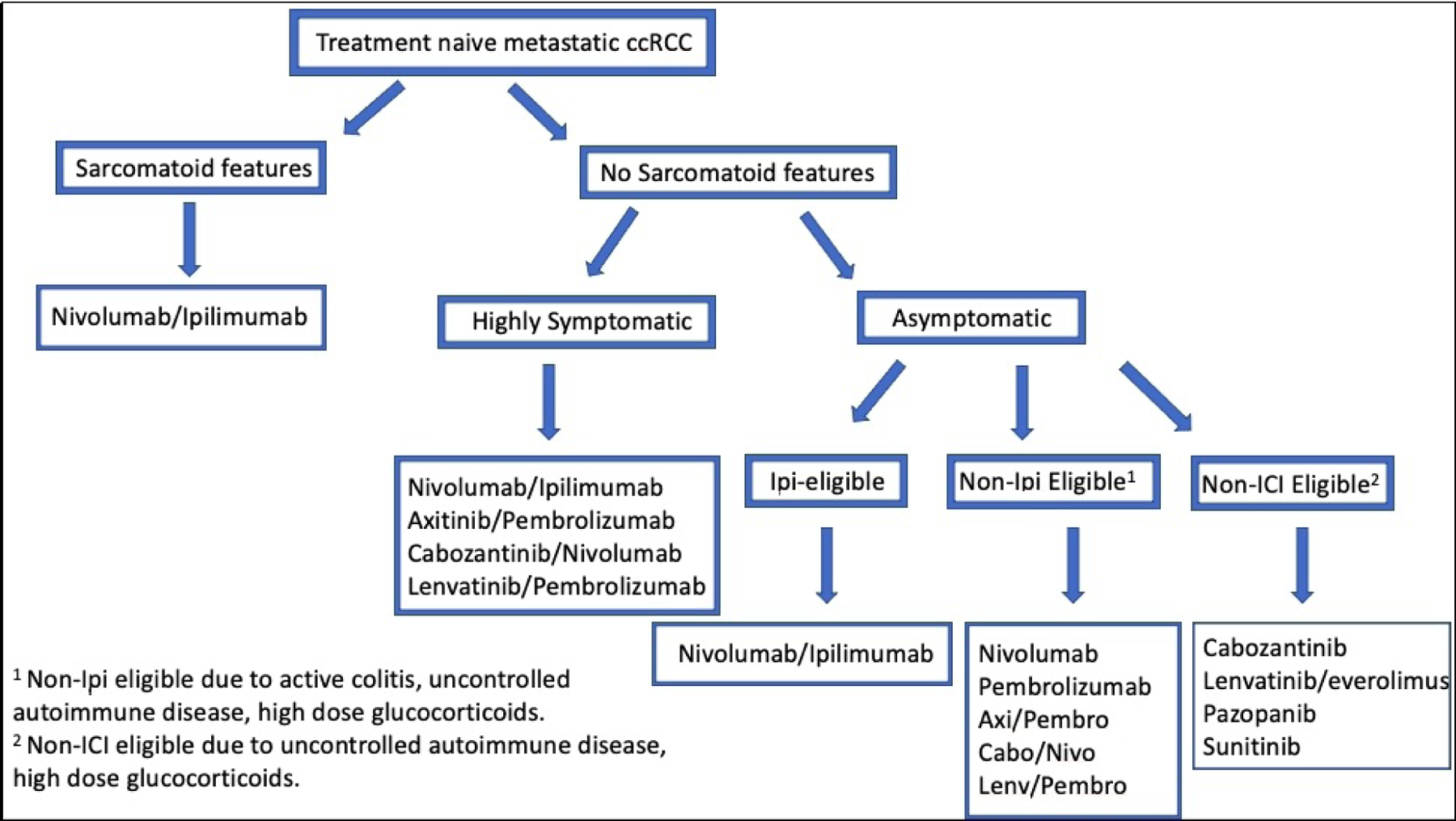
Treatment algorithm for treatmnet naïve metastatic ccRCC. ccRCC: Clear cell renal cell carcinoma.

The first line treatment paradigm for advanced ccRCC has rapidly evolved with an expanding number of combination anti-angiogenic/PD1/L1 regimens including axitinib/pembrolizumab, axitinib/avelumab, cabozantinib/nivolumab and likely lenvatinib/pembrolizumab being added to the existing VEGFR TKI monotherapy and ICI combination regimens. Figure 1 depicts a current treatment algorithm for frontline therapy of patients with advanced ccRCC. All of these anti-angiogenic/anti-PD1/L1 combinations have been (or will be) approved based on benefits relative to sunitinib. These promising early responses must be contextualized to the long-term benefits of dual immune checkpoint blockade with nivolumab/ipilimumab. Indeed, the question of whether antiangiogenic and ICI therapies provide synergistic, additive or sub-additive effects on long-term outcomes, such as cure rate, treatment free survival and landmark PFS and OS remains under intense scrutiny. Surveillance of long-term toxicities and quality of life measures will also be important considerations. Prospectively validated biomarkers will be essential with the potential to match individual patients’ disease biology with checkpoint inhibitors, antiangiogenic TKI, or novel therapies to guide first line and sequential treatment strategies.

## Figures and Tables

**Table 1.** Study design and outcomes from key studies

Study design/treatment	Treatment arms	ORR, CR (by IRC unless otherwise noted)		PFS (by IRC unless otherwise noted)		OS	

		ORR% (CR)	HR (95%CI)	Median, mo	HR (95%CI)	Median, mo	HR (95%CI)
Checkmate 214^[16,43]^ - R, ph 3; treatment naïve patients stratified by IMDC risk (*N* = 1096) - Co-Primary Endpoints: PFS, ORR, OS in int/poor risk - Nivo/Ipi (3 mg/kg Nivo + 1 mg/kg Ipi q3wk for 4 doses, then 3 mg/kg Nivo q2wk) *vs*. Sun (50 mg qd)a	Int/Poor risk Nivo/Ipi (*N* = 425) Sun (*N* = 422)	42.1% (10.1) 26.3% (1.4)	*P* < 0.001	11.6 8.3	0.75 (0.62–0.90) (*P* = 0.015)	47 26.6	0.66 (0.55–0.80) (*P* < 0.0001)
	Favorable risk Nivo/Ipi (*N* = 125) Sun (*N* = 124)	28.8% (12.8) 54.0% (5.6)	*P* < 0.0001	17.0 28.8	1.65 (1.16–2.35) (*P* = 0.0049)	NR NR	1.19 (0.77–1.85) (*P* =0.43)
	ITT Nivo/Ipi (*N* = 550) Sun (*N* = 546)	39.1% (10.7) 32.6% (2.4)	*P* = 0.02	12.4 12.3	0.88 (0.75–1.04) (*P* = 0.126)	NR 38.4	0.72 (0.49–0.95) (*P* = 0.0002)
	Int/Poor (PD-L < 1) Nivo/Ipi (*N* = 284) Sun (*N* = 278)	37% 28%	P = 0.03	11.0 10.4	1.00 (0.8–1.26)	NR NR	0.73 (0.56–0.96)
	Int/Poor (PD-L > 1) Nivo/Ipi (*N* = 100) Sun (*N* = 114)	58% 22%	*P* < 0.001	22.8 5.9	0.46 (0.31–0.67)	NR NR	0.45 (0.29–0.71)
IMmotion 151^[58]^ - R, ph 3; treatment naïve patients (*N* = 915) - Co-Primary Endpoints: PFS (by inv) in PD-L1+, OS in ITT - Atezo/Bev (Atezo 1200 mg IV q3wk + 15 mg/kg Bev IV q3wk) *vs*. Sun (50 mg qd)a	PD-L1+ Atezo/Bev (*N* = 178) Sun (*N* = 184)	Inv 43% (9) 35% (4)	-	Inv 11.2 7.7	0.74 (0.57–096) *P* = 0.02	34.0 32.7	0.84 (0.62–1.15) *P* = 0.28
	PD-L1 + Atezo/Bev (*N* = 178) Sun (*N* = 184)	IRC 36% (15) 33% (8)	-	IRC 8.9 7.2	0.93 (0.72–1.21)	-	-
	ITT Atezo/Bev (*N* = 454) Sun (*N* = 461)	Inv 37% (5) 33% (2)	-	Inv 11.2 8.4	0.83 (0.70–0.97) *P* = 0.02	33.4 34.9	0.93 (0.76–1.14) *P* = 0.47
KEYNOTE 426^[59,60]^ - R, open label ph 3; treatment naïve patients (*N* = 861) - Co-Primary Endpoints: PFS (by ICR), OS in ITT - Axi/Pembro (Axi 5 mg PO qd + Pembro 200 g IV q3wk) *vs*. Sun (50 mg qd)a	ITT Axi/Pembro (*N* = 432) Sun (*N* = 429)	60% (9) 40% (3)	*P* < 0.0001	15.4 11.1	0.71 (0.60–0.84) *P* < 0.0001	NR 35.7	0.68 (0.55–0.85) *P* = 0.0003
	Int/Poor risk Axi/Pembro (*N* = 294) Sun (*N* = 298)	55.8% (8) 35.2%	-	12.7 8.3	0.69 (0.56–0.84) *P* = 0.0002	NR 28.9	0.63 (0.50–0.81) *P* = 0.0001
	Favorable Risk Axi/Pembro (*N* = 138) Sun (*N* = 131)	69.6% (11) 50.4% (6)	-	20.8 18.0	0.79 (0.57–1.09) *P* = 0.078	NR NR	1.06 (0.6–1.86) *P* = 0.58
JAVELIN Renal 101^[61,62]^ - R, ph 3; treatment naïve patients (*N* = 886) - Independent primary endpoints: PFS (by inv) in PD-L1+, OS in PD-L1+ - Axi/Avel (Axi 5 mg PO qd + Avel 10 mg/kg IV q2wk+ *vs*. Sun (50 mg qd)a	PD-L1 + Axi/Avel (*N* = 270) Sun (*N* = 290)	BICR 55.9% (5.6) 27.2% (2.4)	OR = 3.389 (2.34–4.90)	BICR 13.8 7.0	0.62 (0.49–0.77) *P* < 0.0001	NR 28.6	0.83 (0.59–1.15) *P* = 0.13
	ITT Axi/Avel (*N* = 442) Sun (*N* = 444)	BICR 52.5% (3.8) 27.3% (2.0)	OR = 2.99 (2.23–3.99)	BICR 13.8 8.4	0.69 (0.57–0.82) *P* < 0.0001	NR NR	0.80 (0.61–1.02) *P* = 0.039
Checkmate 9ER^[63]^ - R, open label ph 3; treatment naïve patients (*N* = 651) - Primary endpoints: PFS (BICR) in ITT - Cabo/Nivo (Cabo 40 mg PO qd + Nivo 240 mg IV q2wk *vs*. Sun (50 mg qd)a	ITT Cabo/Nivo (*N* = 323) Sun (*N* = 328)	55.7% (8) 27.1% (5)	*P* < 0.0001	16.6 8.3	0.51 (0.41–0.64) *P* <0.0001	NR NR	0.60 (0.40–89) *P* = 0.001
CLEAR^[29]^ - R, open label ph 3; treatment naïve patients (*N* = 1069) - Primary Endpoint: PFS (by IRC) in ITT - Lenv/Pembro (Lenv 18 mg PO qd + Pembro 200 mg IV q3wk *vs*. Lenv 14mg PO qd + Evero 5mg Po qd *vs*. Sun (50mg qd)a	ITT Lenv/Pembro (*N* = 454) Lenv/Evero (*N* = 461) Sun (*N* = 461)	71% (16) 53% (10) 36% (4)	*P* < 0.001 *P* < 0.001	23.9 14.7 9.2	0.39 (0.32–0.49) P < 0.001 0.65 (0.53–0.80) *P* < 0.001	NR^b^ NR^b^ NR^b^	0.66 (0.49–0.88) *P* = 0.005 1.15 (0.88–1.5) *P* = 0.30
**a 4 wk on/2 wk off.**							

Atezo: Atezolizumab; Avel: avelumab; Axi: axitinib; Bev: bevacizumab; Cabo: cabozantinib; CI: confidence interval; Evero: everolimus; HR: hazard ratio; IMDC: International Metastatic Renal Cell Carcinoma; int: intermediate; inv: investigator; Ipi: ipilimumab; IRC: independent review committee; ITT: intention to treat; Lenv: lenvatinib; mo: month(s); NA: not applicable; Nivo: nivolumab; NR: not reached; ORR: objective response rate; OS: overall survival; PD-L1: programmed cell death ligand 1; PFS: progression free survival; Ph: phase; q3wk: every 3 weeks; qd: once daily; r: randomized; Sun: sunitinib; wk: week.

**Table 2.** Hazard ratio over time in key studies

	Checkmate 214^[16,43]^					
	Favorable risk		Intermediate/poor risk		ITT	
Median follow-up	PFS (HR)	OS (HR)	PFS (HR)	OS (HR)	PFS (HR)	OS (HR)
25.2 mo	2.18 (1.29–3.68) (*P* <0.001)	1.45 (0.51–4.12) (*P* =0.27)	0.82 (0.64–1.05) (*P* = 0.03)	0.63 (0.44–0.89) (*P* < 0.001)	0.98 (0.79–1.23) (*P* = 0.85)	0.68 (0.49–0.95) (*P* < 0.001)
32.4 mo	1.23 (0.9–1.69) (*P* = 0.19)	1.22 (0.73–2.04) (*P* = 0.44)	0.77 (0.65–0.90) (*P* < 0.01)	0.66 (0.54–0.80) (*P* < 0.0001)	0.85 (0.73–0.98) (*P* = 0.03)	0.71 (0.59–0.86) (*P* < 0.01)
43.6 mo	1.65 (1.16–2.35) (0.0049)	1.19 (0.77–1.85) (*P* = 0.43)	0.75 (0.62–0.90) (*P* = 0.015)	0.66 (0.55–0.80) (*P* < 0.0001)	0.88 (0.75–1.04) (*P* = 0.126)	0.72 (0.61–0.86) (*P* = 0.0002)
	**Keynote 426** ^[59,60]^					
	**Favorable risk**		**Intermediate (int)/poor risk**		**ITT**	
Median follow-up	PFS (HR)	OS (HR)	PFS (HR)	OS (HR)	PFS (HR)	OS (HR)
12.8 mo	0.81 (0.53–1.24)	0.64 (0.24–1.68)	0.70 (0.54–0.91) (int) 0.58 (0.35–0.94) (poor)	0.53 (0.35–0.82) (int) 0.43 (0.23–0.81) (poor)	0.69 (0.57–0.84) *P* < 0.001	0.53 (0.38–0.74) *P* < 0.001
30.6 mo	0.79 (0.57–1.09) (*P* = 0.078)	1.06 (0.6–1.86) *P* = 0.58	0.69 (0.56–0.84) (*P* = 0.0002)	0.63 (0.5–0.81) (*P* = 0.0001)	0.71 (0.60–0.84) (*P* < 0.0001)	0.68 (0.55–0.85) *P* = 0.0003
	**Javelin Renal 101** ^[61,62]^					
	PD-L1+		ITT			
Median follow-up	PFS (HR)	OS (HR)	PFS (HR)	OS (HR)		
11.6 mo	0.61 (0.47–0.79) *P* < 0.001	0.82 (0.53–1.28) *P* = 0.38	0.69 (0.56–0.84) (*P* < 0.01)	0.69 (0.56–0.84) (*P* < 0.001)		
19.2 mo	0.62 (0.49–0.77) (*P* = 0.0001)	0.83 (0.59–1.15) (*P* = 0.13)	0.69 (0.57–0.82) (*P* < 0.0001)	0.80 (0.61–1.02) (*P* = 0.03)		

HR: Hazard ratio; PFS: progression free survival; ITT: intention to treat; OS: overall survival; PD-L1: programmed cell death ligand 1; mo: month(s).

**Table 3.** Safety data from key studies

	Checkmate 214^[43]^		IMmotion 151^[58]^		KEYNOTE 426^[59,60]^		JAVELIN renal 101^[61,62]^		Checkmate 9ER^[63]^		CLEAR^[29]^		

	Nivo/Ipi ( *N* = 547)	Sun (*N* = 535)	Atezo/Bev ( *N* = 454)	Sun (*N* = 461)	Axi/Pembro ( *N* = 432)	Sun (*N* = 429)	Axi/Avel ( *N* = 442)	Sun (*N* = 444)	Cabo/Nivo ( *N* = 323)	Sun (*N* = 328)	Lenv/Pembro ( *N* = 454)	Lenv/Evero ( *N* = 461)	Sun (*N* = 461)
Dose reductions, %	-	53	-	37	20	28	42	43	56	52	68	73	50
AE leading to discontinuation of entire treatment regimen, %	22	13	5	8	7	12	8	13	5.6	17	13	19	14
Treatment-related deaths, *n*	8	4	5	1	4	7	3	1	1	2	4	3	1
Treatment-emergent AE, grades 3–4%^a^	47	64	40	54	67	62	71	71	61	51	82	83	72
Corticosteroids for IRAE, %^a^	29	-	16	-	NR	-	11	-	16	-	NR	-	-
Grade 3/4 AEs, % Hypertension	< 1	16	14	17	21	18	26	17	13	13	28	23	19
Fatigue	4	9	1	5	2	5	4	4	3	5	4	8	4
Diarrhea	4	5	2	4	7	5	7	3	7	4	10	12	5
PPE	0	9	0	9	5	4	6	4	8	8	4	3	4
Weight Loss	-	-	-	-	-	-	3	1	1	0	8	7	< 1
Decreased appetite	1	1	< 1	2	2	< 1	2	1	2	1	4	6	2
Proteinuria	-	-	3	< 1	3	3	-	-	3	2	8	8	3
Grade 3/4 laboratory abnormalities, % Hypothyrodiism	< 1	< 1	< 1	< 1	< 1	0	< 1	< 1	< 1	< 1	1	1	0
Increased lipase	10	7	-	-	-	-	-	-	6	5	13	4	9
Neutropenia	-	-	< 1	4	< 1	7	< 1	8	< 1	4	1	1	6
Anemia	0	4	< 1	4	< 1	3	2	8	2	4	2	4	5
Thrombocytopenia	0	5	1	5	0	5	< 1	6	< 1	5	1	4	6
Increased ALT	5	2	-	-	12	3	6	3	5	2	4	3	2
Increased AST	4	1	-	-	7	2	4	2	3	1	3	2	1

a Listed are adverse events with a possible immune-mediated cause and infusion reactions that occurred during study treatment or within the 90 days thereafter, regardless of attribution to study treatment or immune relatedness by the investigator. AE: Adverse event; ALT: alanine aminotransferase; AST: aspartate aminotransferase; Atezo: atezolizumab; Avel: avelumab; Axi: axitinib; Bev: bevacizumab; Cabo: cabozantinib; Evero: everolimus; Ipi: ipilimumab; Lenv: lenvatinib; Nivo: nivolumab; NR: not reported; PPE: palmar-plantar erythrodysthesia; Sun: sunitinib; -: not reported.

**Table 4.** Current clincial trails for first line ccRCC

Title	Study design	Patients	Regimen	Study details	Key results/completion status
*Systemic therapy*					
Checkmate 8Y8 (NCT03873402)^[48]^	1L, Phase 3	Int/poor risk (*N* = 418)	Nivo Nivo/Ipi	Co-primary endpoint: PFS and ORR by BICR	Estimated study completion: date April 2025
COSMIC 313 (NCT03937219)^[81]^	1L, Phase 3	Int/poor risk (*N* = 840)	Cabo/Nivo Cabo/Nivo/Ipi	Primary endpoint: duration PFS by BICR	Estimated study completion date: March 2025
PD1GREE (NCT03793166)^[82]^	1L, Phase 3	Int/poor risk (*N* = 1046)	Nivo/Ipi induction, Nivo Nivo/Cabo	Primary endpoint: OS	Estimated study completion date: April 2022
***Surgery/radiation***					
NORDIC-SUN (NCT03977571)^[77]^	1L, Phase 3	Int/poor risk (*N* = 400)	Nivo 3mg/kg/Ipi 1mg/kg q3wk x4 - Delayed CRN/Nivo - Nivo	Primary endpoint: OS	Estimated study completion date: September 2025
CYTOSHRINK (NCT04090710)^[78]^	1L, Phase 2	Int/poor risk (*N* = 78)	Nivo 3 mg/kg/Ipi 1mg/kg q3wk x4 - SBRT prior to cycle 2 - no SBRT	Primary endpoint: PFS	Estimated study completion date: April 2022
Cyto-KIK trial (NCT04322955)^[79]^	1L, Phase 2	Treatment naive (*N* = 48)	Nivo 480 mg q4wk/Cabo 40 mg qd - Delayed CRN	Primary endpoint: % of participants with a complete response	Estimated study completion date: February 2022
PROBE trial (NCT04510597)^[80]^	1L, Phase 3	Treatment naïve (*N* = 364)	Initial CRN then systemic therapy Systemic therapy alone	Primary endpoint: OS	Estimated study completion date: July 2033

Cabo: Cabozantinib; CRN: cytoreductive nephrectomy; IMDC: International Metastatic Renal Cell Carcinoma; int: intermediate; Ipi: ipilimumab; Nivo: nivolumab; ORR: objective response rate; OS: overall survival; PFS: progression free survival; Ph: phase; q3wk: every 3 weeks; qd: once daily; wk: week; SBRT: stereotactic body radiation therapy.

## Data Availability

Not applicable.
